# Dual Mechanisms of Translation Initiation of the Full-Length HIV-1 mRNA Contribute to Gag Synthesis

**DOI:** 10.1371/journal.pone.0068108

**Published:** 2013-07-05

**Authors:** Anne Monette, Fernando Valiente-Echeverría, Matias Rivero, Éric A. Cohen, Marcelo Lopez-Lastra, Andrew J. Mouland

**Affiliations:** 1 HIV-1 Trafficking Laboratory, Lady Davis Institute at the Jewish General Hospital, Montréal, Québec, Canada; 2 Department of Medicine, Division of Experimental Medicine, McGill University, Montreal, Quebec, Canada; 3 Laboratorio de Virología Molecular, Instituto Milenio de Inmunología e Inmunoterapia, Centro de Investigaciones Médicas, Escuela de Medicina, Pontificia Universidad Católica de Chile, Santiago, Chile; 4 Laboratory of Human Retrovirology, Institut de recherches cliniques de Montréal, Montréal, Quebec, Canada; 5 Department of Microbiology and Immunology, McGill University, Montreal, Quebec, Canada; Queensland Institute of Medical Research, Australia

## Abstract

The precursor group-specific antigen (pr55^Gag^) is central to HIV-1 assembly. Its expression alone is sufficient to assemble into virus-like particles. It also selects the genomic RNA for encapsidation and is involved in several important virus-host interactions for viral assembly and restriction, making its synthesis essential for aspects of viral replication. Here, we show that the initiation of translation of the HIV-1 genomic RNA is mediated through both a cap-dependent and an internal ribosome entry site (IRES)-mediated mechanisms. In support of this notion, pr55^Gag^ synthesis was maintained at 70% when cap-dependent translation initiation was blocked by the expression of eIF4G- and PABP targeting viral proteases in two *in vitro* systems and in HIV-1-expressing cells directly infected with poliovirus. While our data reveal that IRES-dependent translation of the viral genomic RNA ensures pr55^Gag^ expression, the synthesis of other HIV-1 proteins, including that of pr160^Gag/Pol^, Vpr and Tat is suppressed early during progressive poliovirus infection. The data presented herein implies that the unspliced HIV-1 genomic RNA utilizes both cap-dependent and IRES-dependent translation initiation to supply pr55^Gag^ for virus assembly and production.

## Introduction

Translation initiation of most eukaryotic mRNAs occurs by a scanning mechanism whereby the 40S ribosomal subunit is recruited to the vicinity of the 5′-cap-structure, a 7-methyl-guanylic acid residue located at the 5′ terminus of eukaryotic mRNAs, and scans in the 5' to 3' direction until an initiation codon is encountered [1], [2], [3]. The 40S ribosomal subunit is recruited to the mRNA as part of the 43S initiation complex, including the eIF2-GTP/Met-tRNAi (initiator tRNA), eIF1A, eIF1 and eIF3 [1], [2], [3]. eIF4F, a multi-subunit complex important for the recruitment process is composed of eIF4E, eIF4A and eIF4G. EIF4E directly binds the 5'-cap structure, while eIF4A, participates by unwinding secondary structure in the 5'-untranslated region (5′UTR) of the mRNA. eIF4G serves as a scaffold for the coordinated assembly of the translation initiation complex as it exhibits binding sites for eIF4A, eIF4E and eIF3. Therefore, eIF4G association with the mRNA cap (via eIF4E) and the 40S ribosomal subunit (via eIF3) leads to the attachment of the template mRNA to the translation machinery [1], [2], [3]. EIF4G also binds the poly(A)-binding protein, PABP, promoting mRNA circularization by coordinating interactions between the 5′-cap structure and the 3′-poly(A) tail of the mRNA (i.e., 5′-cap:eIF4E:eIF4G:PABP:3′-poly(A)), and this coordinated circularization synergistically stimulates mRNA translation.

Cap-dependent translation and mRNA circularization is targeted during viral infection and can be abolished by the expression of different viral proteases [4], [5]. For example, the FMDV L protease cleaves both eIF4GI and eIF4GII but not PABP [6], [7], while the Poliovirus 2A protease cleaves all of them [8]. Retroviral proteases cleave eIF4GI [9]. The HIV-1 protease (PR) cleaves eIF4GI and PABP [10].

Studies on translation initiation of the uncapped picornavirus mRNA have revealed an alternative mechanism of translation initiation in which the recruitment of the initiation complex is mediated by an internal ribosome entry site (IRES) [1], [2], [3]. Functionally, the IRES was identified by inserting the poliovirus or the encephalomyocarditis virus (EMCV) 5′UTR into the intercistronic spacer of a bicistronic construct encoding two proteins [11], [12]. Expression of the second cistron documented the ability of the inserted sequence to promote internal ribosome binding and translation independent of the first cistron [13]. Subsequent studies showed that this alternative mechanism of translation initiation was not restricted to picornaviruses as mRNAs from other virus families including several members of the *Retroviridae* also exhibited IRES-dependent translation initiation [2], [14].

The capped and polyadenylated full-length genomic RNA of the human immunodeficiency virus type 1 (HIV-1) initiates protein synthesis via either the canonical cap-dependent or the IRES-dependent mechanism [15], [16], [17], [18]. The HIV-1 genomic RNA harbours two IRESes: one in the 5′ UTR (the HIV-1 IRES) [16], [17], [19] and a second within the *gag* open reading frame (the 40K-IRES) [18], [20], [21]. The 5′ cap structure and the HIV-1 IRES are expected to drive translation initiation of the viral structural proteins pr55^Gag^ and pr160^Gag/Pol^ [15], [16], [17], [18], [20], [21], while the 40K-IRES is expected to direct translation of the structural proteins and of a novel 40K-Gag isoform of yet unknown function [20], [21]. The potential redundancy of cap- and IRES-dependent mechanisms that drive the synthesis of the viral structural proteins suggests that regulating translation initiation of the HIV-1 mRNA is essential during the viral replication cycle.

Our current understanding of the regulation of HIV-1 mRNA translation initiation remains limited as most of the assays used to evaluate the process have relied on artificial RNA constructs. Several studies support the existence of a functional HIV-1 IRES in the 5′ untranslated region [16], [17], [19], [22], [23], [24] or in the *gag* coding region [20], [21] but other groups have proposed that cap-dependent initiation is the only means to initiate translation of the *gag* mRNA [15], [25] [reviewed in [18], [26]]. These different views, and the knowledge that HIV-1 infection induces cellular conditions unfavorable to cap-dependent translation [10], [16], [27], [28], [29], prompted us to evaluate the synthesis of HIV-1 proteins under conditions in which cap-dependent translation initiation is abrogated. We first programmed protein synthesis with *in vitro* synthesized RNA to show that the complete cleavage of eIF4G, resulting from the *in vitro* expression of the foot and mouth disease virus (FMDV) L protease, only moderately impacted the translation of a monocistronic mRNA harboring the HIV-1 5′UTR. However, translation initiation driven by the HIV-1 5′UTR in the context of a bicistronic RNA is not affected in these conditions – confirming that both cap-dependent and independent mechanisms of translation initiation are at play. We extended these findings by examining if *de novo* HIV-1 protein synthesis, in the context of an infectious proviral clone, was influenced by a progressive poliovirus infection that elicits a rapid shut-down of global cap-dependent translation. The results from this study revealed, for the first time, that IRES-dependent translation ensures abundant expression of pr55^Gag^. There was, however, a striking block to the *de novo* synthesis of other viral proteins including pr160^Gag/Pol^, Vpr and Tat during poliovirus infection. Thus, during HIV-1 replication, translation initiation from the HIV-1 full-length genomic RNA can occur by dual mechanisms, including both cap- and/or IRES-dependent mechanisms.

## Materials and Methods

### Plasmids

**Figure 1:**
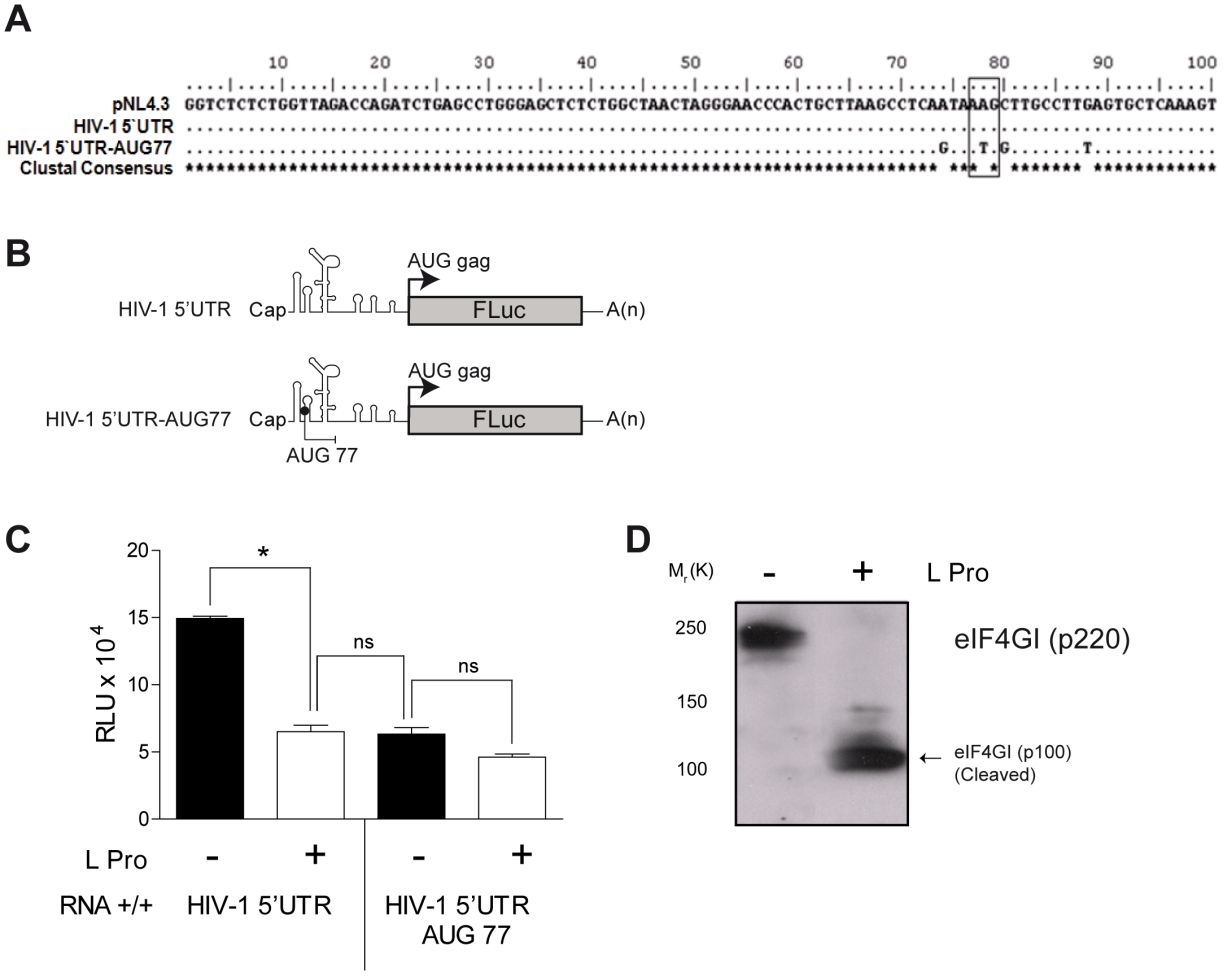
Contribution of the HIV-1 IRES to the overall translational activity displayed by the capped HIV-1 5′UTR. (A) The partial sequence of the HIV-1 5′UTR from the proviral clone pNL4.3 (AF324493l) was aligned against the intercistronic region, HIV-1 5′UTR, recovered from the dl HIV-1 IRES plasmid [16] and the mutant HIV-1 5′UTR-AUG 77 in which an optimal initiation codon was inserted at position 77 with respect to the reference sequence. The block highlights the introduced initiation codon. (B) Schematic representation of the capped and polyadenylated monocistronic mRNAs used in this study. In the HIV-1 5′UTR monocistronic RNA the 5′UTR of the full-length HIV-1 mRNA was cloned upstream of the FLuc reporter. The reporter open reading frame (ORF) was left in frame with the Gag protein initiation codon (arrow). In the HIV-1 5′UTR AUG 77 monocistronic RNA and additional optimal initiation codon has been added at position 77 upstream of the putative internal ribosome entry site (IRES) and out of frame (end point line) with respect to the FLuc ORF. (C) *In vitro* transcribed capped and polyadenylated (+/+) RNA were translated in absence (−; black bar) or presence (+; white bar) of FMDV L protease [33]. Values are the means ± SEM from three independent experiments each conducted in triplicate. (D) Analysis of eIF4GI cleavage. RRL translation reactions (10 ul) without (lane 1) or with (lane 2) FMDV L protease (2% V/V) were resolved by SDS/gradient 5%–20% PAGE, transferred to nitrocellulose membrane, detected using polyclonal antibodies eIF4GI as previously described [33]. Statistical analysis was performed by Student *t*-test (*p<0.05). (B).

The dlΔEMCV and dl HIV-1 IRES plasmids have been previously described [16], [23], [30]. Mutations (AUG 77) were introduced in the HIV-1 5′UTR of the proviral clone pNL4.3 by overlapping extension PCR [31]. In brief, the HIV-1 5′UTR-FLuc was amplified from the dl HIV-1 IRES template vector using two different sets of primers (set 1: 5′-TTTGAAAAACAC*GAATTC*GGTCTCTCTG-3′ and 5′- AAGGCAA*C* C*A*TTA*C*TGAGG-3′; set 2: 5′- CG*TCTAGA*ATTACACTGCGATCTTTCCG-3′ and 5′- CCTCA*G*TAA*T*G *G*TTGCCTT-3′). Both PCR products were denatured, mixed and amplified using the external set of primers to regenerate the full-length HIV-1 5′UTR-FLuc region harboring the AUG77 mutations within the HIV-1 5′UTR. The amplicon was cloned into the pGEM™-T Easy vector (Promega) to generate the pGEM-AUG77-FLuc plasmid. The mutated HIV-1 5′UTR was then recovered by digesting the pGEM-AUG77-FLuc with EcoRI and NcoI and inserting it into the intercistronic region of dl HIV-1 IRES plasmid as described [16], [23], previously digested with the same enzymes (Fermentas). Upon sequencing, an additional mutation (G88T shown in Figure 1A), not originally included in the primers, was identified.

### *In vitro* Transcription

Capped RNAs were synthesized using the mMESSAGE mMACHINE High Yield Capped RNA Transcription Kit (Ambion) and polyadenylated using the Poly(A) Tailing Kit (Applied Biosystems/Ambion), according to the manufacturer's protocol. Upon synthesis, RNAs were treated with DNAse RQ1 (Promega) for 20 min at 37°C. RNA was precipitated with 2.5 M LiCl, centrifuged at 16000×*g* for 30 min at 4°C, washed with 70% ethanol and resuspended in 50 µl of nuclease-free water [23], [24]. RNA concentrations were determined by spectrophotometry (NanoDrop Technology, Wilmington, Delaware, USA) and RNA integrity was monitored by electrophoresis on denaturing agarose gels [23].

### *In vitro* Translation

*In vitro* transcribed dlHIV-1 IRES RNAs (8 ng/µl) were translated in 35% (v/v) nuclease-treated rabbit reticulocyte lysate (RRL; Promega) supplemented with G2/M HeLa cells extracts and salts as previously described [18], [23]. For the FMDV L protease assays, uncapped-RNA encoding the FMDV L protease generated from the pLb plasmid (a kind gift of Graham Belsham, Institute for Animal Health, Pirbright, UK) was linearized with Xba I (Fermentas) [32], and was *in vitro* transcribed in nuclease-treated RRL as previously described [33]. Briefly, 220 ng/µl of the FMDV L protease uncapped-RNA was translated in 35% (v/v) of RRL for 90 min. The L protease-RRL was diluted in fresh RRL 35% (v/v) in a ratio of 1∶2 and 1∶4. The diluted L protease-RRL was added to fresh RRL as described above and was used at a final concentration of 4% (v/v) for the translation reaction [33]. Translation reactions were incubated at 30°C for 90 min. Luciferase activities were measured using the DLR™ Assay System (Promega) according to manufacturer’s instructions on a Sirius Luminometer (Berthold Detection Systems GmbH, Pforzheim, Germany) as previously described [23], [30], [33].

### Cell Culture and Plasmid Transfections

HEK293T [34] (see below) and HeLa [22] cells were grown in Dulbecco's modified Eagle's medium (Invitrogen) supplemented with 10% heat-inactivated fetal bovine serum (HyClone) at 37°C in a humidified atmosphere containing 5% CO_2_. HeLa cells were seeded at 6×10^5^ cell/well in 6-well plates and were infected for 1 hr with the recombinant vaccinia virus vTF7-3 (which expresses the T7 RNA polymerase) 24 hrs later [35]. Next, the plasmids (2 µg pNL4-3, 500 ng dlHIV-1 IRES [16] and 1 µg pLb [32], [36]) were co-transfected and cells were collected at 24 hrs post-transfection and processed for western analysis and/or IRES activity determinations.

### Western Analysis and IRES Activity Determinations

For western analysis, cells were washed with PBS (pH 7.5) and lysed in NTEN buffer (100 mm NaCl, 10 mm Tris, pH 7.5, 1 mm EDTA, 0.5% Nonidet P-40, Roche Protease inhibitor, and 10 U RNaseOUT (Invitrogen)). Cell lysates were quantified for protein content by the Bradford assay (Bio-Rad) and equal quantities of protein were loaded onto SDS-polyacrylamide gels. Proteins were then transferred to nitrocellulose membranes (Pall). Blocked membranes were incubated with primary antibodies of interest, and then with horseradish peroxidase-conjugated secondary antibodies (Rockland). Chemiluminescence detection was performed using the Western Lightning Chemiluminescence Reagent Plus kit (Perkin-Elmer Life Sciences), according to the manufacturer's instructions. For IRES activity determinations, cells were harvested with passive lysis buffer (Promega) and 20 µg of cellular protein (as measured using the Bradford assay (BioRad)) was used to assess IRES activity with the dual-luciferase reporter assay system according to the manufacturer's instructions (Promega). To calculate IRES activity, IRES-driven Firefly luciferase (Fluc) expression was normalized to cap-dependent Renilla luciferase (Rluc) activity, as described previously [22], [23], [30], [33]. EIF4GI cleavage by the FMDV L protease was assessed by 4–20% gradient SDS-PAGE of RRL (10 µl; re: Figure 1) followed by transfer to nitrocellulose membranes (Pall). Protein abundance was quantified on film by densitometry using the ImageJ software (http://rsbweb.nih.gov/ij/index.html). Each experiment designed to test IRES activity was performed in triplicate in three independent experiments.

### Antibodies

Mouse anti-p24 and goat anti-gp120 were provided by the National Institutes of Health AIDS Reference and Reagent Program and goat anti-eIF3η was purchased from Santa Cruz Biotechnology. Rabbit anti-eIF4GI and anti-eIF4GII antibodies were provided by Nahum Sonenberg (McGill University, Canada); anti-G3BP1 was provided by Imed Gallouzi (McGill University, Canada); anti-PABP was obtained from Sigma-Aldrich and anti-GAPDH from Techni-Science, Inc (Montreal, Canada). A human serum from an HIV-1 infected patient, No. 162 [37] was used for immunoprecipitation to identify multiple HIV-1 proteins. This antiserum, as well as the pre-immune human serum used in the study identified multiple poliovirus antigens since the donors were vaccinated against poliovirus. For subsequent immunoprecipitations, rabbit anti-Tat (No. R1.3) and rabbit anti-Vpr (No. R3.7) antisera were used at 1∶200, as described [34], [38].

### HIV-1/poliovirus Transfection/infection and Immunoprecipitation Experiments

pNL4.3 proviral DNA [39] and/or dlHIV-1 IRES plasmid were transfected into 293T cells [34] and at 24 hr post-transfection, cells were washed and infected with the Mahoney strain of poliovirus type 1 at a m.o.i. of 5–10 using a Poliovirus stock (1×10^9^ plaque-forming units (pfu)/mL) as described [36] and which was kindly provided by Yuri Svitkin and Nahum Sonenberg (McGill University, Canada). pcDNA3.1 DNA was used in mock transfections and in mock transfection/infection experiments with poliovirus. Cell-free poliovirus stocks were prepared by *de novo* infection of 293T cells followed by three freeze-thaw cycles. Titres were determined empirically to provide maximal inhibition of *de novo* protein synthesis at about 3–4 hrs post-infection (>80% inhibition of total protein synthesis), when cleavage of eIF4GI, eIF4GII, PABP and maximal inhibition of host protein synthesis occur (Supplemental Figure S1). Total protein synthesis was determined by TCA precipitation as described [40]. Poliovirus (10–20 c.f.u./mL) was incubated in serum- and Met/Cys-free Dulbecco’s modified Eagle medium (DMEM) for 1 hr 200 µL with constant rotation. Fresh, FCS-containing and pre-warmed medium was then added. At the indicated time-points following infection (0–6 hrs), cells were pulsed with 400–450 µCi/mL [^35^]S-Trans-Label radiolabelled amino acids (Amersham) for 20 minutes (1 Ci = 37 GBq) at 37°C in Met/Cys amino-acid-free DMEM, before harvesting and processing for immunoprecipitation analyses and western blotting. Cells were washed extensively and lysed in 1X RIPA buffer (150 mM NaCl, 0.5% NP-40, 0.1%SDS, 0.5% deoxycholate (DOC), 10 mM Tris-HCL, pH 7.5, 1 mM EDTA). All cell lysates were centrifuged (15 000×*g*, 30 min, 4°C) and then pre-cleared using pre-immune normal human and rabbit serum and 50 µL 50∶50 slurry of protein G Sepharose (Thermo-Scientific) for 2 hrs with constant rotation, as described [34]. Pre-cleared supernatants were transferred to fresh tubes containing antibodies against viral proteins described above and as indicated in figure legends. Following overnight incubation, Protein G Sepharose was added for 1 hr and three washes were then performed with IP Buffer (25 mM Tris, 150 mM NaCL; pH 7.2). Sequential immunoprecipitations were performed with anti-HIV-1 (No. 162) first, followed by other viral antigen-specific antisera (either anti-Vpr or anti-Tat). Pre-immune (for pre-clearing) and immune sera was used for mock immunoprecipitations for all radiolabelling experiments to identify non-specific signals in autoradiographs. Immunoprecipitates were separated in 4–20% gradient SDS-PAGE gels. Gels were fixed with 40% methanol/10% acetic acid, treated with En3Hance (Perkin-Elmer) and processed for autoradiography. The results presented are representative of experiments that were reproduced two to five times.

### Statistical Analysis

The statistical data analysis and graphics described in the text were done using the GraphPad v5.03 program (La Jolla, CA 92037, USA). Differences were tested by Student *t*-test where p<0.05 was considered significant. BioEdit v7.0.9 (Ibis Biosciences, Carlsbad, CA 92008, USA) was used for sequence alignments and analysis.

## Results and Discussion

Controversy exists regarding the mechanism used by the HIV-1 genomic mRNA to initiate protein synthesis during an infection cycle [15], [16], [17], [18]. In the context of a bicistronic mRNA isolated from any other viral protein or RNA sequence, the 5′UTR of the HIV-1 genomic RNA is capable of programming IRES-dependent translation initiation [16], [17]. However, studies conducted using monocistronic mRNAs mimicking the full-length viral mRNA challenged the requirement of an IRES for Gag synthesis [15], [18].

To evaluate whether HIV-1 IRES activity, originally described in the context of a bicistronic mRNA [16], [17], [19], contributes to the overall translational activity of the capped HIV-1 5′UTR we designed the monocistronic HIV-1 5′UTR and HIV-1 5′UTR-AUG77 RNAs (Figure 1A–1B). The HIV-1 leader (nucleotide position 1–336 with respect to pNL4.3) of these RNAs was placed immediately 5′ of the firefly luciferase reporter gene (FLuc). The HIV-1 5′UTR-AUG77 RNA was designed to uncouple cap-dependent from IRES-driven translation initiation using a previously described strategy [15]. For this purpose, an additional initiation codon in an optimal context, defined by a purine at position (–3) and guanine at position (+4) (GAUAUGG) [41], but out of frame with respect to the FLuc initiation codon was introduced in the HIV-1 5′UTR. The first position of the introduced initiation codon corresponds to nucleotide 77 with respect to the numbering of pNL4.3 clone (where position 1 corresponds to the initiation of the R region of the viral LTR; AF324493; Figure 1B). The rationale behind introducing this additional initiation codon at position 77 considers that the minimal HIV-1 IRES activity has been described between nucleotides 104 and 336 (with respect to the pNL4.3 clone) [16]. Should the translation initiation complex be recruited in a cap-dependent fashion, it would be expected that scanning 40S ribosomal subunits would recognize AUG77 as the initiation codon. Thus, the most important feature of this system is that the translation product from the artificially generated upstream open reading frame (uORF) does not encode a functional FLuc reporter protein. As a consequence, FLuc activity from the HIV-1 5′UTR-AUG77 RNA can only be expected to represent IRES driven translation initiation.

In the first series of experiments, we asked whether the capped HIV-1 5′UTR and HIV-1 5′UTR-AUG77 monocistronic mRNAs could drive FLuc synthesis. *In vitro*-generated transcripts encoding the HIV-1 leader and HIV-1 5′UTR-AUG77 were translated in rabbit reticulocyte lysate (RRL) supplemented with Hela extracts as described in Materials and Methods [23]. The monocistronic HIV-1 5′UTR RNA exhibited higher FLuc activity (RLU: 15.0±0.17) than that obtained for HIV-1 5′UTR-AUG77 RNA (RLU: 6.32±0.47) (Figure 1C, black bars). That the HIV-1 5′UTR-AUG77 RNA does allow FLuc expression suggests that the HIV-1 IRES also contributes to overall protein synthesis (Figure 1C, black bars). Alternatively, this result could also imply that the AUG77 is somehow bypassed by scanning 40S ribosomal subunits, leaky scanning mechanism, or that upon translation of the small ORF generated by the inclusion of AUG77 in the 5′UTR, the 40S ribosomal subunits initially recruited by a cap-dependent mechanism might reinitiate translation though a termination-reinitiation mechanism [2]. In both alternative scenarios translation would initiate at the downstream AUG allowing FLuc expression. Noteworthy, the termination-reinitiation mechanism of translation has been recently proposed to explain the initiation from the 5′UTR of the HIV-1 mRNA when additional initiation codons are added upstream of the *gag* AUG [15]. Based on these possibilities, we next decided to conduct similar *in vitro* translation experiments in the presence of the foot and mouth disease virus (FMDV) L protease [2], [16], [18]. The FMDV L protease cleaves eIF4G [7], [36], [42], inactivating the eIF4F complex in terms of its ability to recognize capped mRNAs and hence results in the arrest of cap-dependent translation initiation [6], [43]. The carboxyl-terminal fragment of eIF4G generated by FMDV L protease cleavage is however, able to recruit eIF3 and the 40S ribosome to efficiently support cap-independent, IRES-mediated translation initiation [44]. In this experimental setting, if expression of the FLuc reporter is indeed driven by a cap-dependent mechanism, such as leaky scanning or termination-reinitiation, then expression of FLuc would be strongly attenuated when translation is conducted in the presence of the FMDV L protease. On the other hand, previous studies have shown that HIV-1 IRES activity is resistant to eIF4G cleavage by FMDV L [16], [33]. Therefore, this simple experimental approach was used to discriminate between the possible initiation mechanisms used to drive FLuc expression in the context of the HIV-1 5′UTR-AUG77 monocistronic RNA. For these experiments, the FMDV L protease was prepared in RRL and 4% v/v of RRL-L protease was added to fresh RRL supplemented with G2/M HeLa extracts, as previously described [33]. Cleavage of eIF4GI as monitored by immunoblotting, was shown to be almost complete in this *in vitro* setting (Figure 1D) [33], [36], [45]. In the presence of the FMDV L protease, translation from the capped HIV-1 5′UTR RNA was reduced 2.3-fold in comparison without FMDV L protease (RLU:6.5±0.47 v/s 15.0±0.17), while translation of the capped HIV-1 5′UTR-AUG77 RNA was not significantly affected in comparison without FMDV L protease (RLU: 4.6±0.22 v/s 6.32±0.47) (Figure 1C, white bars). This result, which is not consistent with either the leaky scanning or a termination-reinitiation model, confirms that FLuc expression from the capped HIV-1 5′UTR-AUG77 RNA is mediated by a IRES-dependent mechanism. Strikingly, the FLuc activity from the capped HIV-1 5′UTR RNA in the presence of FMDV L protease was comparable to that of the lone reporter activity from the capped HIV-1 5′UTR-AUG77 RNA (Figure 1C, compare middle bars). This observation suggests that translation initiation from the capped HIV-1 5′UTR RNA can be driven by both the cap-structure and the IRES as inhibition of cap-dependent translation by eIF4G cleavage does not abrogate FLuc synthesis. Though experiments using the capped HIV-1 5′UTR RNA clearly show that IRES activity accounts for FLuc synthesis in the presence of FMDV L protease, they do not allow us to conclude whether the capped HIV-1 5′UTR RNA is exclusively translated using a cap-dependent mechanism or if the mRNA is constitutively expressed using both cap- and IRES-dependent mechanisms to initiate translation. One other possibility that remains to be examined is whether inhibition of cap-dependent translation initiation by the addition of an upstream AUG or eIF4G cleavage, induces a switch to IRES-mediated translation initiation – a mechanism that has been described for other mRNAs that initiate via a dual cap-dependent and IRES-dependent mechanisms [46], [47], [48], [49], [50].

**Figure 2:**
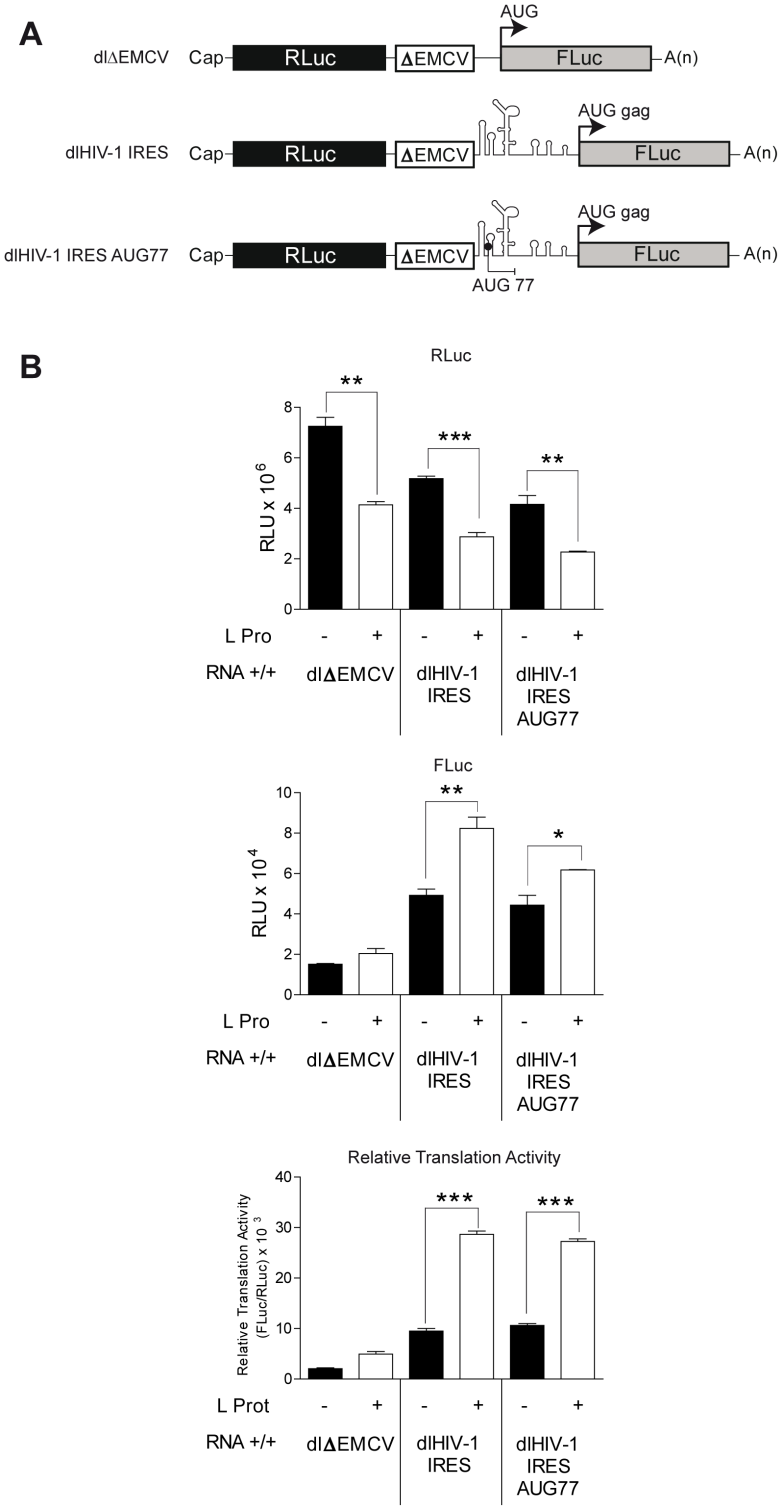
FMDV L protease negatively impacts on the translation of cap-dependent translation but not on translation initiation mediated by the downstream IRES element. (A) Schematic representation of the capped and polyadenylated bicistronic mRNAs used in this study. Vectors dlΔEMCV and dl HIV-1 IRES have been previously described [16], [70]. The 5′UTR recovered from the HIV-1 infectious clone pNL4.3, dl HIV-1 IRES, was mutated as described in figure 1 to add an additional initiation codon in an optimal context at position 77 (with respect to pNL4.3), AUG 77. The mutated HIV-1 5′UTR was cloned into dual luciferase bicistronic (dl) vectors harboring RLuc and FLuc luciferase as reporter genes to generate the dl HIV-1 IRES AUG 77 vector. The 5′UTR recovered from the HIV-1 infectious clone pNL4.3, dl HIV-1 IRES, was used as the reference IRES [16], while the RLuc/FLuc bicistronic vector harboring a defective encephalomyocarditis virus (ΔEMCV) IRES, dlΔEMCV, was used as a negative control [16], [70]. As indicated by the arrows and dead end lines the AUG 77 initiation codon is out of frame with respect to the FLuc ORF. (B) *In vitro* transcribed capped and polyadenylated (+/+) RNA were translated in absence (−; black bar) or presence (+; white bar) of FMDV-L protease [33]. RLuc (left panel) and FLuc (middle panel) activities, as well as the relative translation activity (right panel) which corresponds to the [(FLuc/RLuc)] ratio, normally used as an index of IRES activity, are shown. Values are the means ± SEM from three independent experiments each conducted in triplicate. Statistical analysis was performed by Student *t*-test (*p<0.05; **p<0.01; ***p<0.001).

To further validate the data presented above, the HIV-1 leader region containing mutation AUG77 was next evaluated in the context of a dual luciferase (dl) reporter construct containing upstream *Renilla luciferase* (RLuc) and downstream FLuc genes (Figure 2A). In this new experimental setup, FLuc activity strictly depends on IRES expression driven by the HIV-1 5′ leader sequence [16], [17] [19]. These experiments were conducted in both the presence and absence of FMDV L protease, which, as above, decreased the translation of cap-driven RLuc from the first cistron of these dl RNAs (Figure 2B, top graph), and increased the synthesis of IRES-driven Fluc (Figure 2B, middle graph). A similar behavior was previously reported for the dl HIV-1 IRES mRNA [16], [33]. Normalized FLuc activities for both bicistronic mRNAs were comparable (see relative translation efficiency expressed as the FLuc/RLuc ratio in Figure 2, bottom graph), confirming that the inclusion of an additional AUG within the HIV-1 5′UTR at position 77 does not affect HIV-1 IRES-mediated translation initiation.

**Figure 3:**
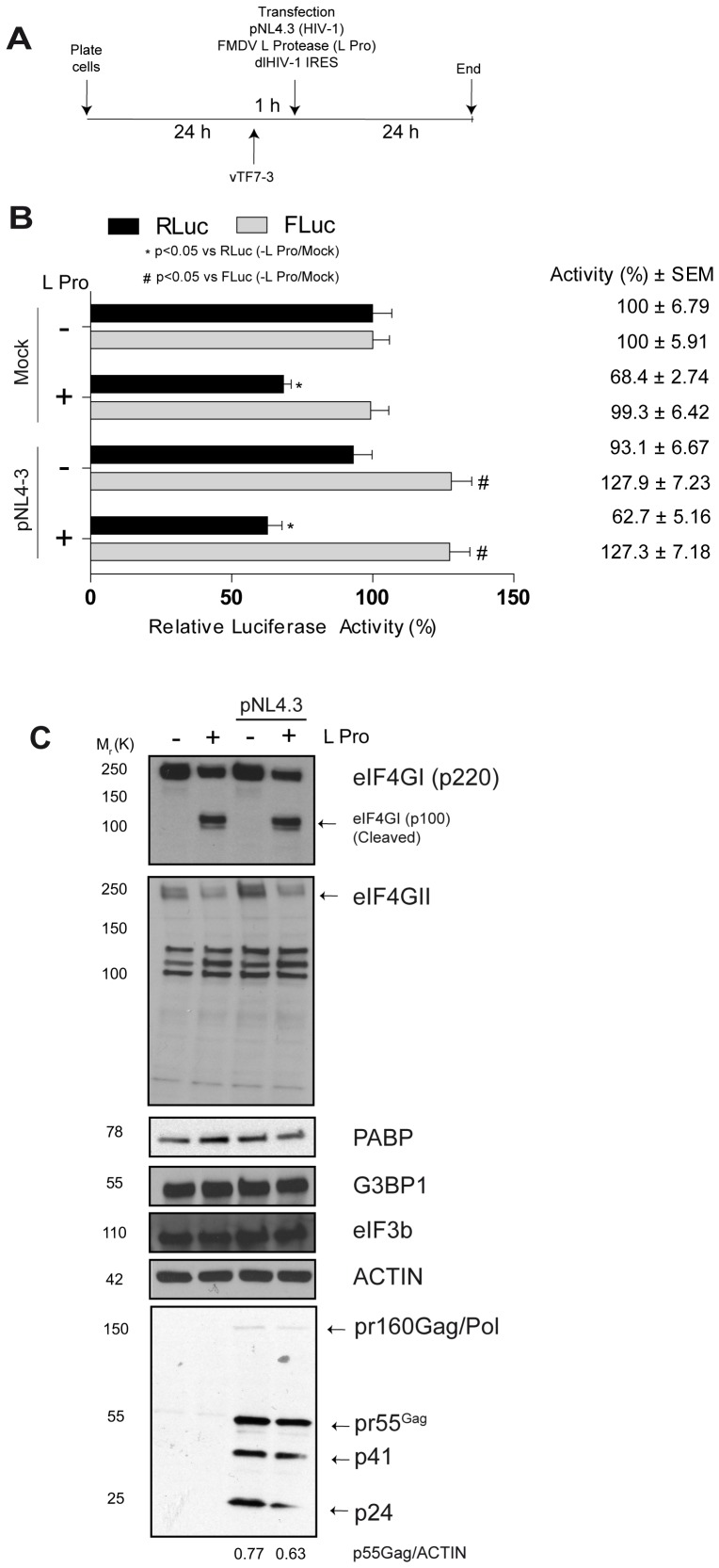
Examination of HIV-1 IRES expression and expression levels of viral proteins during the expression of FMDV L protease in cells. (A) Schematic representation of the experimental protocol. (B). IRES activity levels were determined as described in Materials and Methods. RLuc and FLuc activities of the dl HIV-1 IRES in the uninfected “mock” cells in the absence of FMDV L protease were arbitrarily set to 100%. Statistical analysis was performed by the Student’s *t*-test (p<0.05) where (*) represents a significant difference v/s RLuc activity in uninfected “mock” cells in the absence of L Pro, while (^#^) represents a significant difference v/s FLuc activity in uninfected “mock” cells in the absence of L Pro (C). Whole cell lysates were analyzed for host eIF4GI, eIF4GII, PABP, G3BP1, eIF3b, Actin and pr55^Gag^ by western blotting analyses.

Since the above experiments were performed in an *in vitro* setting, we sought to determine if IRES-dependent translation initiation would be favoured or conserved during the expression of the FMDV L protease in live cells expressing HIV-1. In these experiments, cells were infected with recombinant vaccinia virus vTF7-3 and were then co-transfected with a DNA expression vector encoding a T7 RNA Polymerase-driven FMDV L protease (L Pro), the dlHIV-1 IRES construct, and mock or pNL4.3 plasmid (i.e., encoding the full complement of HIV-1 genes) (Figure 3A). Luciferase activity was measured following cell harvesting at 24 hrs, and results were expressed as relative luciferase activity. Mock cells in the absence of L pro were arbitrarily set to 100% (± SEM). Cap-dependent translation activity was found to be inhibited by 32% (compare the black bars in uninfected “mock” cells in the absence or presence of L Pro, Figure 3B) while the HIV-1 IRES activity was unaffected in the presence of the FMDV L protease (compare the grey bars in mock cells in the absence or presence of L Pro, Figure 3B). Recapitulating our previous findings [22], co-expression of HIV-1 (pNL4-3) with dlHIV-1 IRES construct enhanced IRES-driven translation by 27.9%, suggesting that the cellular environment created from HIV-1 expression favours IRES-mediated mRNA translation (compare the grey bars in mock and transfected (pNL4-3) cells in the absence of L Pro, Figure 3B). Next, co-expression of HIV-1, dlHIV-1 IRES construct and the FMDV L protease still enhanced HIV-1 IRES activity (compare the grey bars in transfected (pNL4.3) cells in the absence or presence of L Pro, Figure 3B) while cap-dependent translation initiation was decreased by 37% (compare the black bars in transfected (pNL4.3) cells in the absence or presence of L Pro, Figure 3B). Again, these data suggest that translation initiation driven by the HIV-1 5′UTR is maintained under conditions known to block cap-dependent translation (Figure 1). To validate this, we evaluated how the expression of the FMDV L protease impacted on the steady-state levels of other cellular and viral proteins. T7-induced expression of the FMDV L protease resulted in a partial cleavage of both eIF4GI and eIF4GII (Figure 3C). The partial cleavage of eIF4G may be the result of lower than expected RNA stability of T7-driven transcripts due to inefficient 5′ capping [51]. There was no effect on steady-state expression levels of PABP, G3BP1, eIF3b or Actin due to the expression of the viral protease. Interestingly, when HIV-1 was co-expressed in the presence of the FMDV L protease, steady-state levels of pr55^Gag^ were not modulated significantly despite the cleavage of eIF4G in cells (Figure 3C). Even though not fully conclusive due to the partial cleavage of eIF4GI and eIF4GII these data suggest that in the context of a replicating viral clone, where cap-dependent translation initiation is targeted by FMDV L protease, pr55^Gag^ synthesis is maintained, most likely due to IRES-mediated translation initiation of the HIV-1 genomic RNA. Based on our earlier published reports, the effects of the proteases on protein stability was negligible [16], [18], [33], and our results favour the notion that pr55^Gag^ synthesis is not significantly affected by the FMDV L protease suggesting the presence of a functional IRES within the HIV-1 genomic RNA.

The later steps of the HIV-1 replication cycle are mainly directed by the precursor, group-specific antigen, pr55^Gag^. It drives virion assembly and is sufficient for the organization, budding and release of virus-like particles (VLPs) from cells [52]. Pr55^Gag^ also selects the HIV-1 genomic RNA from the cytoplasm for encapsidation [53], [54] and during virus maturation, is cleaved to give rise to several structural proteins [55]. In addition to pr55^Gag^, other viral proteins and numerous host proteins are required for the assembly of infectious HIV-1 particles [56]. Following synthesis, pr55^Gag^ is thought to rapidly associate to membranes for targeting to viral assembly sites [39], [57], [58] with the concerted activities of motor and adaptor proteins [59], [60], [61], [62]. Therefore, it is conceivable that the virus has engineered the translation of its genomic RNA to ensure adequate expression of pr55^Gag^ throughout the viral replication cycle.

**Figure 4:**
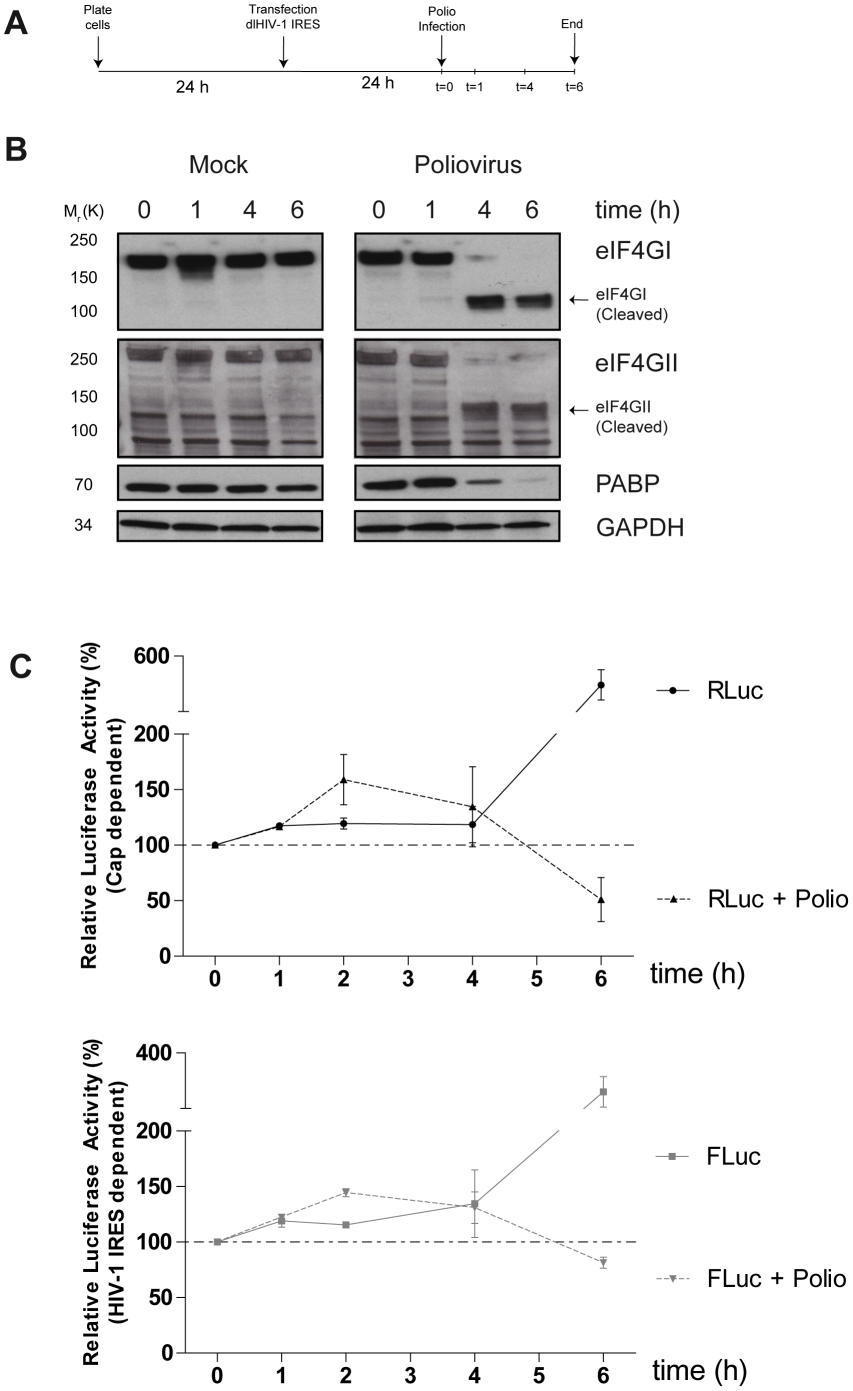
Poliovirus infection does not inhibit HIV-1 IRES activity in cells. (A) Schematic representation of the experimental protocol. (B). Whole cell lysates were analyzed for host proteins eIF4GI, eIFGII and PABP by western blotting analyses. GAPDH served as a loading control. (C). RLuc (top panel) and FLuc (bottom panel) activities over the course of poliovirus infection are shown. Luciferase activities at *t = 0* (before poliovirus infection) were set to 100%. Values represent the means ± SD from two independent experiments.

Earlier work showed that the genomic RNA could be translated when cap-dependent translation is suppressed during poliovirus (PV) infection and when critical translation factors are cleaved [20]. This earlier study only examined Gag expression levels however, and little else was shown on the expression levels of the other HIV-1 gene products and the relative contributions of cap-dependent and independent translation mechanisms for HIV-1 expression. To address this issue, we next evaluated the contributions of the HIV-1 IRES activity in transfection-infection experiments. To do this, dl HIV-1 IRES DNA was transfected into 293T cells that were then infected with poliovirus 24 hr later (Figure 4A). The poliovirus titre used to infect the cells was chosen such that there was an 80–90% shut down of total protein synthesis in the overall culture. The required poliovirus titres were determined in empirical metabolic labeling studies (Supplemental Figure S1). At several time points post-poliovirus infection, cells were collected to perform western blot analysis and measure luciferase activity. Poliovirus induced rapid cleavage of translation initiation factors eIF4GI, eIF4GII, and PABP (Figure 4B; [36], [63]), drastically inhibiting cap-dependent translation as reflected by relative RLuc activity (compare *t = 0*: 100%; *t = 6*: 51%) (Figure 4C, top). Moreover, we found that translation of HIV-1 IRES-driven FLuc was not significantly impacted by poliovirus infection (compare *t = 0*: 100%; *t = 6*: 81.5%) (Figure 4C, bottom). These results suggest that HIV-1 IRES remains functional during the time course of poliovirus infection.

**Figure 5:**
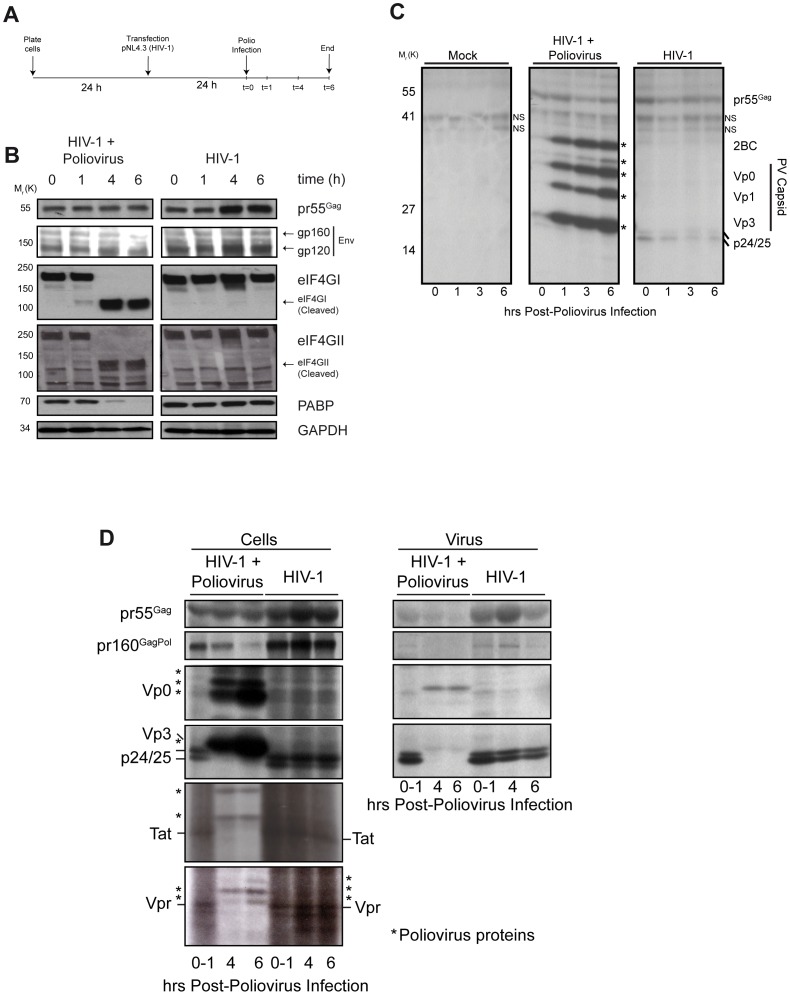
Poliovirus infection does not block HIV-1 pr55^Gag^ synthesis but dramatically reduces 4kb- and 2kb-encoded viral gene products, Vpr and Tat. (A). Schematic representation of the experimental protocol. (B) Whole cell lysates were analyzed for viral pr55^Gag^ and gp160/gp120 (Env) and host proteins eIF4GI, eIFGII and PABP by western blotting analyses. GAPDH served as a loading control (bottom). (C,D) At the time points indicated post-infection, cells were briefly pulsed with radiolabelled amino acids and immediately harvested for immunoprecipitation analysis. Metabolically-labeled proteins found in whole cell lysates (Panel C (cells), Panel D (left panel (cells)) and cell-free virus lysates (Panel D (right panel (virus)) were immunoprecipitated using human anti-HIV-1 No. 162. Cell lysates were sequentially immunoprecipitated with rabbit anti-Tat and anti-Vpr antisera (Panel D, left panel) and revealed by fluorography. Fractionation was performed by SDS-PAGE (either 10 or 14% polyacrylamide gels) as described in Materials and Methods. Non-specific bands are indicated (NS) and the poliovirus Capsid proteins (2BC, Vp0, Vp1, Vp2 & Vp3) and low molecular weight (*) poliovirus proteins found in immunoprecipitates are indicated; molecular weights are indicated on left.

To evaluate the contributions of the cap and the IRES on global HIV-1 gene expression, HIV-1 proviral DNA was transfected into 293T cells that were then infected with poliovirus 24 hr later as shown in Figure 5A. Poliovirus infection induced the complete cleavage of eIF4GI, eIF4GII, and PABP by ∼4 hrs post-infection while the steady-state expression of pr55^Gag^ was stable throughout the time course of the experiment (Figure 5B). As expected, the expression levels of viral proteins such as Env (gp120/160) translated from viral mRNAs that do not harbor IRES sequences [64] were found to be decreased in the presence of poliovirus (Figure 5B).

The continuous presence of pr55^Gag^ during poliovirus infection reported in Figure 5A could be due to protein stability or be the result of its continuous mRNA translation. In view of these possibilities we next the assessed if during poliovirus infection HIV-1 mRNA was indeed translated. In these experiments, *de novo* synthesis of viral proteins was assessed by a brief pulse of radiolabeled amino acids following an identical transfection/infection protocol shown in Figure 5A and as described in Materials and Methods. Cells were harvested, and viral proteins were immunoprecipitated using an infected patient’s serum (No. 162; [37]). This assay highlighted, first, that *de novo* synthesized pr55^Gag^ was relatively stable at all-time points following poliovirus infection (Figure 5C), despite cleavage of eIF4GI, eIF4GII and PABP. The pr55^Gag^ signal was specific, since in mock conditions, immunoprecipitation using the same human serum did not precipitate radiolabelled bands at this molecular weight. While *de novo* synthesis of pr55^Gag^ expression was stable, that of p25/p24 decreased over time, suggesting a possible effect on HIV-1 protease gene expression or activity. The patient’s immune serum identified multiple poliovirus antigens that were evident within 1 hour post-infection and that sharply increased at time points thereafter (Figure 5C, middle panel).

In a similarly designed experiment, we queried how poliovirus-mediated shutdown of cap-dependent translation initiation influences *de novo* synthesis of other viral proteins. The HIV-1 serum also recognizes several precursors and processed HIV-1 antigens including pr160^Gag/Pol^, pr55^Gag^ and p24/25 Capsid [37]. Again, *de novo* synthesis of pr55^Gag^ was maintained at 70% ±10 (SEM) levels at the latest time point post-infection in several experiments, suggesting that the shutdown of cap-dependent translation elicited by poliovirus infection does not abrogate pr55^Gag^ synthesis. *De novo* synthesis of pr160^Gag/Pol^, a translation product of ribosomal −1 frameshifting of the same genomic RNA, however, fell off abruptly and was most evident by 4–6 hrs post-infection (Figure 5D)**.** As this precursor protein encodes the viral protease, p25/24 levels concomitantly disappeared, as did the signals for RT and IN (data not shown; Figure 5C & 5D). These observations cannot be readily explained but one possibility is that the switch from a cap-dependent to an IRES mode of translation alters the RNA structure in such a way that negatively impacts of the RNA conformation required for ribosomal −1 frameshifting. While this possibility is speculative, a recent report on ribosomal −1 frameshifting of a retroviral mRNA also possessing an IRES element revealed that ribosomal −1 frameshifting is highly dependent on an equilibrium between a permissive and non-permissive RNA conformations [65]. Further support for this type of regulation comes from recent work in which a correlation between HIV-1 translation initiation and RNA structure has been proposed [66].

Poliovirus gene products increased dramatically at post-infection times, and following virus purification, a notable appearance of poliovirus proteins was detected in cell-free supernatants with prominent bands for poliovirus Capsid proteins (Figure 5D, right panel). When the expression levels of candidate genes encoded by the HIV-1 singly-spliced and multiply-spliced mRNAs were assessed, we found that the *de novo* expression of Vpr (4 kb) and Tat (2 kb), could not be detected by immunoprecipitation/fluorography analyses following poliovirus infection at 4 hrs post-infection – despite abundant pr55^Gag^ expression (Figure 5D). The signals for these auxiliary and regulatory proteins were specific as determined by the employment of control anti-Vpr and anti-Tat antisera in immunoprecipitation assays from mock expressing cells (data not shown). Thus, poliovirus infection negatively impacts on the synthesis of HIV-1 gene products except that of pr55^Gag^, indicating that the synthesis of HIV-1 pr55^Gag^ is preserved following the poliovirus induced shutdown of cap-dependent RNA translation.

One unexpected finding of this study was the negative impact of poliovirus infection on the expression of the viral protein, Tat. Two recent reports suggest that like the genomic RNA, *tat* mRNA is translated by both cap-dependent and IRES-dependent mechanisms [67] [68]. These recent studies used dual luciferase bicistronic vectors to identify IRES-driven translation mediated by the 5′UTR of the *tat* mRNA whereas this study favoured IRES-mediated translation imposed by PV infection. Because both poliovirus 2A and 3C proteases cleave PABP in a differential polysome-associated manner [63], this experimental situation, in contrast to the dual luciferase experimental system, would lead to the cleavage of PABP (Figure 4 & 5) thereby preventing PABP-mediated mRNA circularization. In addition, PABP cleavage by poliovirus 3C protease inhibits translation from IRESes that require the PABP interaction with the poly(A) tail [69], which could also explain why Tat is not synthesized when PABP is completely cleaved (Figure 5D). Finally, in support of our findings is the observation that pr55^Gag^ synthesis is PABP- and eIF4GI-independent (Figure 5B and [10]).

This work substantiates an IRES-dependent mechanism to initiate HIV-1 genomic mRNA translation. In addition, our data now reveal that the expression of auxiliary and regulatory HIV-1 gene products is sensitive to the block in cap-dependent translation imparted by the poliovirus 2A protease activity, as these were not detectable in progressive poliovirus infection. At this point, we are unable to fully ascertain that the observed inhibition during poliovirus infection is only due to eIF4G cleavage by the poliovirus 2A protease or whether it is a combined effect involving PABP cleavage by poliovirus 2A and poliovirus 3C proteases causing a detrimental effect on global cap-dependent translation. Nevertheless, our data suggest that the HIV-1 IRES drives the synthesis of pr55^Gag^ at a time when cap- and Poly(A)-dependent translation is suppressed.

## Supporting Information

Figure S1 **Titration of the poliovirus inoculum.** Various concentrations of cell-free poliovirus were used to determine the poliovirus inoculum to elicit a maximum effect on total protein synthesis in cells. 293T cells were incubated with increasing quantities of poliovirus inoculum as described in Materials and Methods. Cells were pulsed with >400 µCi ^[35S]^Trans-Label (Amersham) for 2–3 hours in Met- and Cys-free DMEM. Trichloroacetic acid-precipitable counts (TCA) as a measure of protein synthesis were estimated by liquid scintillation counting. B, A 100 µL inoculum was used for subsequent experimentation and this resulted in a maximal decrease in host protein synthesis at 2–4 hr post-infection, coincident with the peak in eIF4G cleavage and cap-dependent translation shut-off induced by poliovirus. C, Cells were infected with poliovirus, pulsed with >400 µCi ^[35S]^TransLabel and the human anti-HIV-1 (No. 162) serum was used to immunoprecipitate viral proteins from cells at 4 hr post-infection. Immunoprecipitates were separated on SDS-PAGE gels were soaked in Enhance (Amersham), dried and then exposed for autoradiography. A typical blot showing radiolabeled poliovirus proteins appearing as a function of time (see main manuscript for Materials and Methods). This is an underexposed gel and HIV-1 proteins have not appeared in this exposure. Similar results were obtained in at least 5 independent determinations.
(TIF)
