# Supplementary figures and images for: Dual Mechanisms of Translation Initiation of the Full-Length HIV-1 mRNA Contribute to Gag Synthesis
Source: PLoS One. 2013 Jul 5;8(7):e68108. doi: 10.1371/journal.pone.0068108 (PMC3702555; doi:10.1371/journal.pone.0068108)

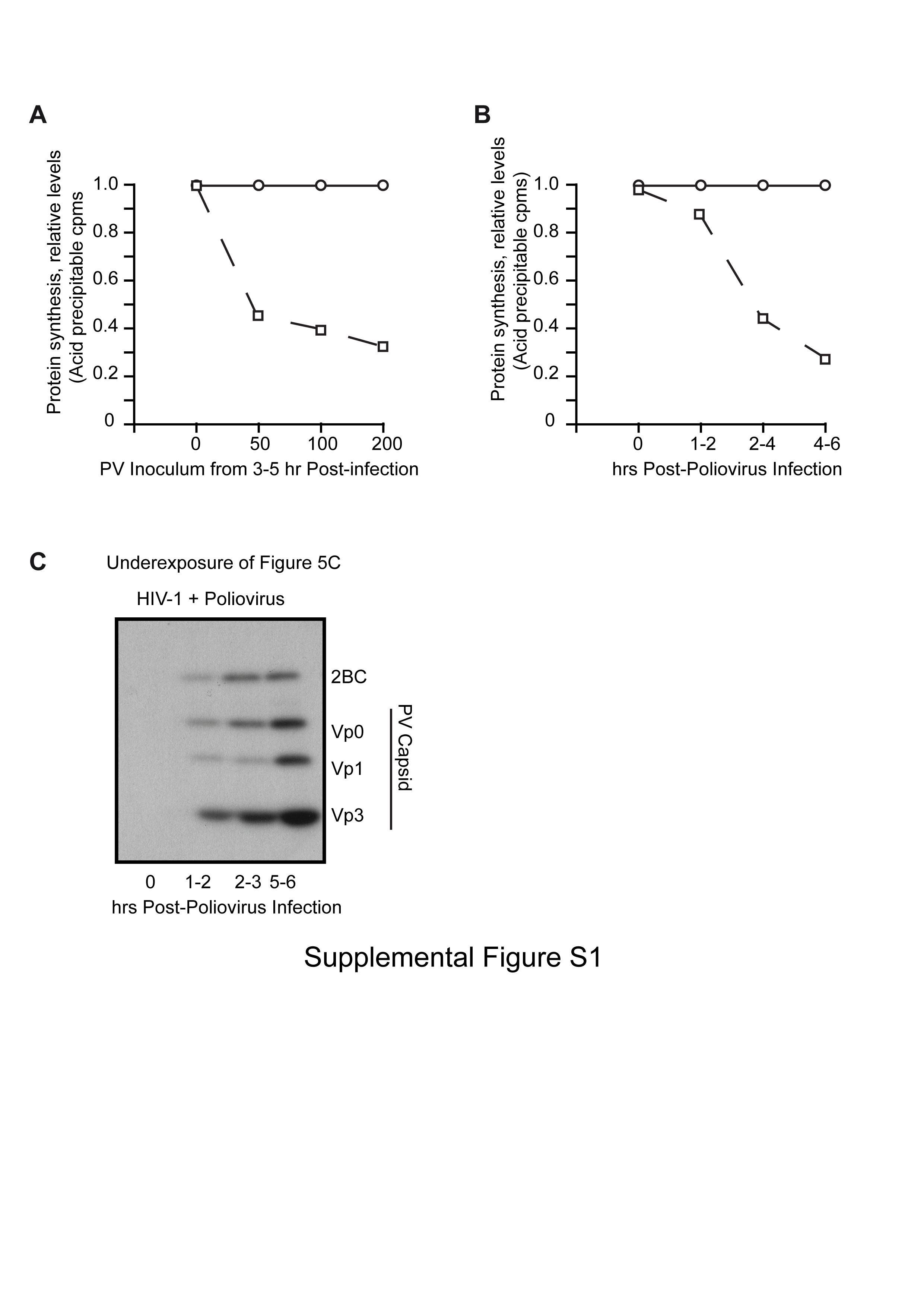

Supplement: Figure S1 — Titration of the poliovirus inoculum. Various concentrations of cell-free poliovirus were used to determine the poliovirus inoculum to elicit a maximum effect on total protein synthesis in cells. 293T cells were incubated with increasing quantities of poliovirus inoculum as described in Materials and Methods. Cells were pulsed with >400 µCi [35S]Trans-Label (Amersham) for 2–3 hours in Met- and Cys-free DMEM. Trichloroacetic acid-precipitable counts (TCA) as a measure of protein synthesis were estimated by liquid scintillation counting. B, A 100 µL inoculum was used for subsequent experimentation and this resulted in a maximal decrease in host protein synthesis at 2–4 hr post-infection, coincident with the peak in eIF4G cleavage and cap-dependent translation shut-off induced by poliovirus. C, Cells were infected with poliovirus, pulsed with >400 µCi [35S]TransLabel and the human anti-HIV-1 (No. 162) serum was used to immunoprecipitate viral proteins from cells at 4 hr post-infection. Immunoprecipitates were separated on SDS-PAGE gels were soaked in Enhance (Amersham), dried and then exposed for autoradiography. A typical blot showing radiolabeled poliovirus proteins appearing as a function of time (see main manuscript for Materials and Methods). This is an underexposed gel and HIV-1 proteins have not appeared in this exposure. Similar results were obtained in at least 5 independent determinations. (TIF) [file pone.0068108.s001.tif]
